# Case Report: Primary epithelioid sarcoma of orbit: first case in Asia and literature review

**DOI:** 10.3389/fonc.2026.1836728

**Published:** 2026-06-26

**Authors:** Guang Yang, Jingping Yuan, Fangfang Chen, Honglin Yan, Wen Liu

**Affiliations:** 1 Department of Pathology, Renmin Hospital of Wuhan University, Wuhan, China; 2 Department of Pathology, People’s Hospital of Luotian County, Huanggang, China

**Keywords:** differential diagnosis, epithelioid sarcoma, immunohistochemistry, INI1, orbital neoplasms

## Abstract

**Background:**

Epithelioid sarcoma is an exceedingly rare malignant soft tissue neoplasm with unclear histogenesis and distinctive epithelioid morphology. Primary epithelioid sarcoma of orbit represents an extremely rare clinical entity, with only isolated case reports documented globally. Notably, no formal, peer-reviewed case report of primary epithelioid sarcoma of orbit has been published in Asia to date.

**Case presentation:**

A 25-year-old male patient presented with slowly progressive, painless proptosis of the left eye. Orbital MRI revealed an irregular mass in the superomedial extraconal space of the left orbit, with an initial radiological impression of vascular tumor or inflammatory lesion. Histologically, the tumor displayed characteristic multinodular growth, composed of a mixture of epithelioid and spindle cells with necrotizing granuloma-like changes. Skeletal muscle invasion and vascular tumor emboli were observed. Immunohistochemically, tumor cells co-expressed epithelial markers (broad-spectrum CK, EMA), mesenchymal marker (Vimentin) and CD34, with loss of nuclear INI1 (SMARCB1) expression. Whole-body PET-CT performed postoperatively excluded distant metastasis, and a diagnosis of classic primary epithelioid sarcoma of the left orbit was confirmed. The patient underwent extended tumor resection combined with local intensity-modulated radiotherapy. No local recurrence or distant metastasis was detected at 7 months postoperatively.

**Conclusions:**

Primary epithelioid sarcoma of the orbit is diagnostically challenging, and pathological examination remains the gold standard for diagnosis. The combination of characteristic histological features and a distinct immunophenotype (loss of INI1) is critical for definitive diagnosis and differential diagnosis. We report the first case of primary epithelioid sarcoma of orbit in Asia, which enriches the clinicopathological data of this rare tumor in the Asian population and provides a key reference for its clinical management and pathological diagnosis. However, as a single-case study, larger series are needed to confirm generalizability.

## 1 Introduction

Epithelioid sarcoma is a malignant soft tissue tumor characterized by epithelioid morphology and incompletely elucidated histogenesis, first systematically characterized by Enzinger in 1970 (1). Accounting for less than 1% of all soft tissue sarcomas, it is classified as an extremely rare neoplasm (2, 3). Based on clinicopathological features, two major subtypes are recognized: the classic (distal) subtype, which predominantly affects superficial soft tissues of the distal extremities in adolescents and young adults; and the proximal subtype, which typically arises in deep soft tissues of the trunk, pelvis and genital region in middle-aged to elderly patients, exhibiting greater aggressiveness and frequent rhabdoid cytomorphology (4, 5).

Primary epithelioid sarcoma of orbit is an exceptionally rare entity arising at an atypical anatomical site. Since the initial case report in 1994 (6), only sporadic cases have been documented globally (6–10), all of which are confined to Europe and the United States. To date, no case of primary epithelioid sarcoma of orbit has been reported in Asia from PubMed and Web of Science (last searched December 2025, keywords: epithelioid sarcoma, orbit; no language limits), resulting in a shortage of relevant clinicopathological data for the Asian population.

Herein, we report a detailed case of classic Primary Epithelioid Sarcoma of Orbit diagnosed in China, representing the first documented case in Asia. Through systematic review and comparative analysis of 6 fully documented cases previously reported in Europe and the United States (6–10), we comprehensively discuss the clinicopathological features, diagnostic pitfalls, research progress and therapeutic strategies of this tumor, to provide a reliable reference for clinical and pathological practice.

## 2 Case presentation

### 2.1 Basic information

**Figure 1:**
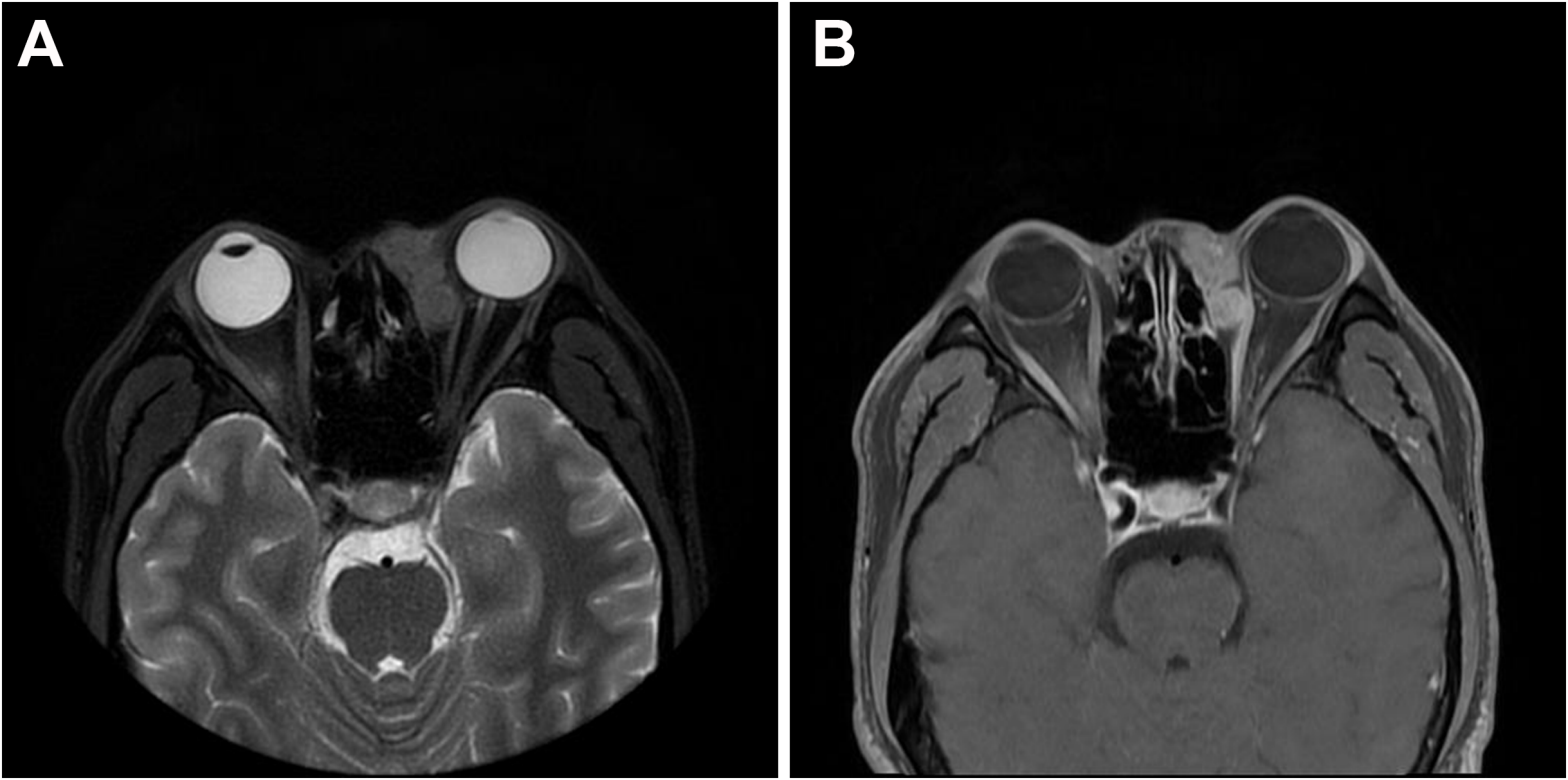
Imaging characteristics of the tumor on MRI. **(A)** Precontrast MRI scan; **(B)** Contrast-enhanced MRI scan.

A 25-year-old male patient was admitted with a painless mass in the left orbit for 1 year and progressive enlargement with proptosis for 3 months. He had no remarkable past medical history. Orbital MRI revealed an irregular mass with isointense signal on T1- and T2-weighted images in the extraconal space of the superomedial quadrant of the left orbit, measuring approximately 2.1 cm × 2.4 cm × 3.4 cm with a clear boundary. Contrast-enhanced scanning showed inhomogeneous fill-in enhancement. The lesion was initially considered to be a vascular tumor or inflammatory lesion (**Figures 1A, B**). The patient underwent extended tumor resection, and the postoperative specimen was sent for pathological examination. Postoperative whole-body PET-CT revealed no evidence of metastatic lesions elsewhere.

### 2.2 Histopathological findings

Gross examination showed grayish-brown fragmented tissue with a total volume of approximately 3.5 cm × 3.0 cm × 1.2 cm. The cut surface was grayish-white with medium texture and locally firm consistency.

**Figure 2:**
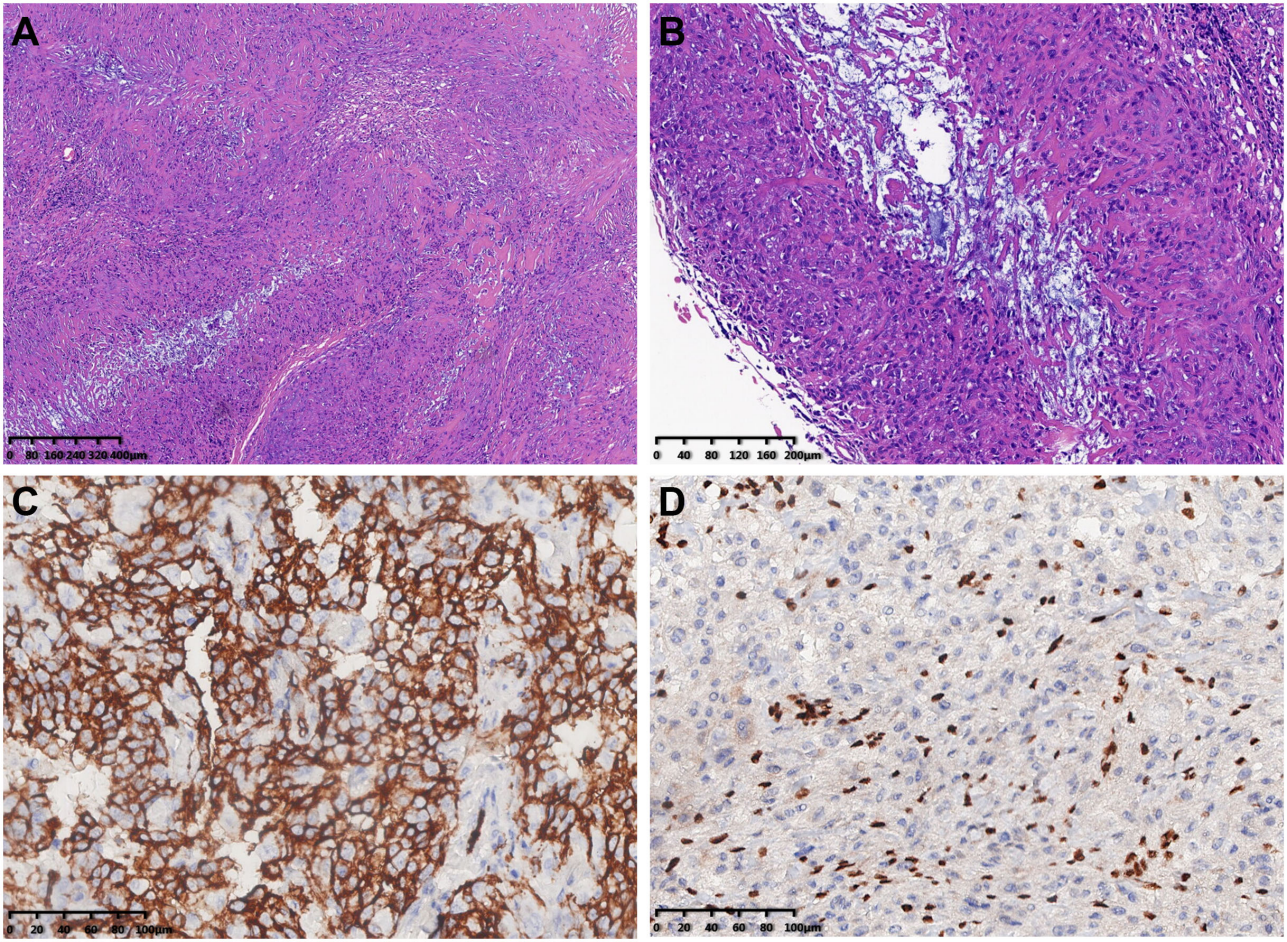
Histopathological features of the tumor. **(A)** Epithelioid cells showing ovoid and plump spindle morphology with abundant cytoplasm, admixed with spindle cells at the periphery. Scale bar, 400 μm. **(B)** Nodular granuloma-like structure composed of epithelioid and spindle cells with central necrosis. Scale bar, 200 μm. **(C)** Tumor cells were positive for CD34. Scale bar, 100 μm. **(D)** Loss of INI1 expression in tumor cells. Scale bar, 100 μm.

Microscopically, the tumor exhibited a multinodular architecture with variable nodule sizes. It was mainly composed of two cell types. The majority were epithelioid cells, round, polygonal or plump spindle-shaped, with abundant cytoplasm and distinct nucleoli. Scattered spindle cells were present at the periphery of the tumor or admixed with epithelioid cells (**Figure 2A**). Some nodules showed granuloma-like structures formed by epithelioid and spindle cells, with focal necrosis (**Figure 2B**). Focal degeneration, hemorrhage, dystrophic calcification, and myxoid change were identified in the stroma, accompanied by scattered infiltration of lymphocytes and histiocytes. Vessels were abundant in some stromal areas.

The tumor invaded adjacent skeletal muscle. Furthermore, tumor emboli were identified within vascular lumens (vascular invasion), and tumor cells surrounded nerve bundles (perineural invasion).

### 2.3 Immunophenotype

Tumor cells co-expressed epithelial markers (CK-pan, EMA) and mesenchymal marker (Vimentin). Most tumor cells were positive for CD34 (**Figure 2C**). The most critical finding was loss of nuclear INI1 (SMARCB1) expression in tumor cells (**Figure 2D**), whereas endothelial cells, lymphocytes and other surrounding normal structures retained intact nuclear staining, serving as an excellent internal control. Tumor cells were negative for CD31, ERG, SSTR2, PR, SOX10, SMA, and MUC4. P53 showed scattered weak positivity. The Ki-67 proliferation index was approximately 20%, indicating moderate proliferative activity. The immunophenotype of this case is shown in **Table 1**.

**Table 1:** Immunohistochemical panel and results in the present case.

Antibody	Clone	Result (tumor cells)	Internal control
CK-pan	AE1/AE3	Positive	–
EMA	E29	Positive	–
Vimentin	V9	Positive	Stromal fibroblasts, endothelial cells, lymphocytes
CD34	QBEnd10	Positive	Endothelial cells
INI1	25(GeneTech)	Negative	Lymphocytes, endothelial cells (nuclear staining retained)
CD31	JC70A	Negative	Endothelial cells
ERG	MXR004	Negative	Endothelial cells
SSTR2	EP149	Negative	–
PR	PgR636	Negative	–
SOX10	EP268	Negative	–
SMA	1A4	Negative	Smooth muscle
MUC4	8G7	Negative	–
p53	DO-7	Scattered weak positive	Normal cells (scattered weak nuclear staining)
Ki-67	SP6	~20%	–

### 2.4 Molecular findings

**Figure 3:**
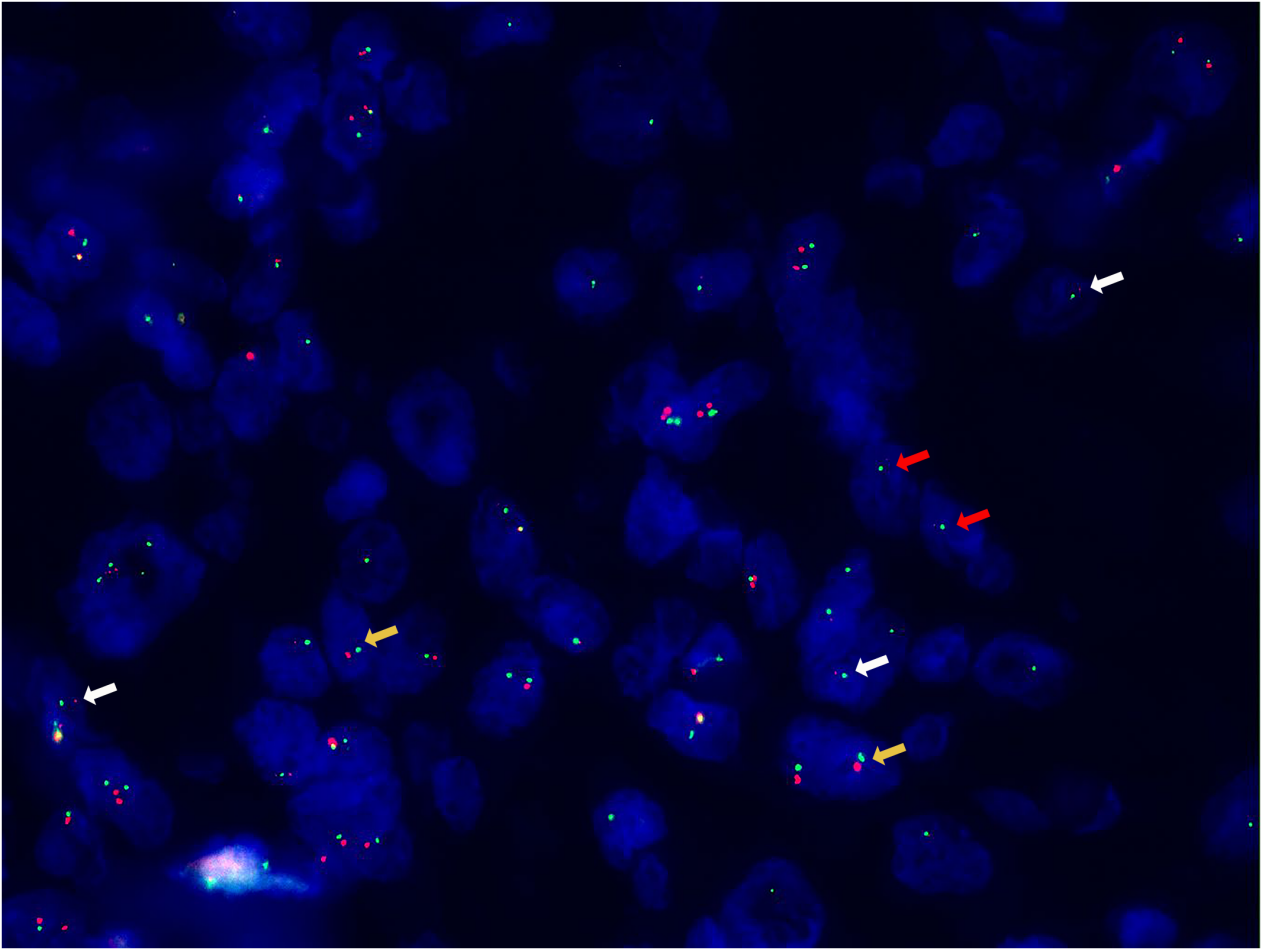
Fluorescence *in situ* hybridization (FISH) analysis of SMARCB1 (22q11) deletion in the present case. FISH was performed using a dual-color probe set: SMARCB1 (22q11, orange signal) and a reference probe at 22q12 (green signal). Magnification: ×1000. Heterozygous deletion (1R1G, yellow arrow) was observed, indicating loss of one *SMARCB1* allele. Partial deletion of a single *SMARCB1* allele (1R[dim]1G, white arrow) was characterized by markedly diminished orange signal intensity, suggestive of intragenic deletion or copy number loss of a fragment. Homozygous deletion (0R1G, red arrow), with complete loss of both orange signals, was observed in a minor subpopulation of tumor cells.

To further characterize the *SMARCB1* gene status, fluorescence *in situ* hybridization (FISH) was performed on formalin-fixed, paraffin-embedded tumor tissue using a dual-color probe set: *SMARCB1* (22q11, orange signal) and a reference probe at 22q12 (green signal). A total of 100 cells were counted. The results showed heterogeneous signal patterns: heterozygous deletion (1R1G) was observed in 18% of cells, partial deletion of one allele with diminished orange signal [1R(dim)1G] in 36%, homozygous deletion (0R1G) in 6%, and normal 2R2G pattern in 30% (**Figure 3**). The presence of a markedly diminished orange signal [1R(dim)1G] suggested a structural partial deletion of one *SMARCB1* allele, such as an intragenic deletion or copy number loss of a fragment.

### 2.5 Pathological diagnosis

Combined with histological morphology and immunophenotype, the final pathological diagnosis was: Classic epithelioid sarcoma of the left orbit. Metastasis from other sites was recommended to be excluded before confirmation of primary orbital origin.

### 2.6 Treatment and follow-up

Whole-body PET-CT was performed postoperatively and showed no evidence of tumor in other sites. To reduce the risk of local recurrence, the patient received adjuvant intensity-modulated radiotherapy (IMRT) with a total dose of 60 Gy. At 7 months of postoperative follow-up, no clinical or radiological evidence of local recurrence or distant metastasis was detected. The patient is scheduled for regular follow-up with clinical examination and orbital imaging every 3 months for the first 2 years, then every 6 months for the next 2 years, and annually thereafter, in accordance with the National Comprehensive Cancer Network (NCCN) Clinical Practice Guidelines for Soft Tissue Sarcoma (11, 12).

## 3 Discussion

Primary orbital epithelioid sarcoma represents a diagnostic challenge for both clinicians and pathologists. Its clinical manifestations overlap with common benign orbital tumors or inflammatory disorders, and its histomorphological features can be easily confused with various malignant orbital neoplasms. Due to its extremely low incidence and limited awareness, misdiagnosis and missed diagnosis occur frequently in clinical practice.

To our knowledge, the present case represented the first pathologically confirmed case of Primary epithelioid sarcoma of orbit in Asia. Combined with the clinicopathological features of this case and a comprehensive analysis of 6 previously reported cases worldwide (**Table 2**) (6–10), we discussed the clinical characteristics, pathological diagnosis, differential diagnosis, treatment modalities and prognostic factors of this rare entity, aiming to provide a practical reference for the diagnosis and management of this tumor.

**Table 2:** Summary of reported cases of primary epithelioid sarcoma of the orbit.

Case	Year	Age/sex	Histological subtype	Main treatment	Outcome	Country
White et al. (6)	1994	34 years/F	Classic	Surgical resection	Died of pelvic metastasis 29 months after diagnosis	Canada
White et al. (6)	1994	17 years/F	Classic	Surgical resection	Disease-free survival at 3 years postoperatively	Canada
Alkatan et al. (7)	2011	5 months/M	Proximal	Tumor debulking + chemotherapy	Died of intracranial invasion 9 months after diagnosis	Saudi Arabia
Thranitz et al. (8)	2014	30 years/M	Proximal	Orbital exenteration + chemotherapy	Died of bone metastasis 14 months after diagnosis	Germany
Jurdy et al. (9)	2016	39 years/F	Classic	Wide excision + adjuvant radiotherapy	Disease-free survival at 5 years postoperatively	Netherlands
Kaya et al. (10)	2018	87 years/F	Proximal	Orbital exenteration + adjuvant radiotherapy	Favorable outcome at 3-month follow-up	United States
Present case	2025	25 years/M	Classic	Extended resection + adjuvant radiotherapy	No recurrence or metastasis at 7-month follow-up	China

### 3.1 Clinical features

Reported patients exhibited a wide age distribution, ranging from a 5-month-old infant to an 87-year-old woman, with a median age of approximately 34 years. The male-to-female ratio was roughly 3:4, indicating no obvious sex predilection. The most prevalent clinical presentation was progressive, painless or painful proptosis, which could be accompanied by diminished visual acuity and diplopia. Due to the non-specific clinical symptoms, all cases were preoperatively suspected to be other more common orbital lesions, such as inflammatory pseudotumor, hemangioma, lymphoproliferative disorders, or even metastatic carcinoma. Our patient presented with painless proptosis, and preoperative MRI also suggested hemangioma. This finding highlighted the limitations of clinical and imaging-based diagnosis, and definitive diagnosis relied entirely on histopathological examination. The MRI features of an irregular, well-defined, inhomogeneously enhancing mass were consistent with the multinodular architecture and focal necrosis identified histologically.

### 3.2 Pathological features

The histological characteristics of orbital epithelioid sarcoma were identical to those of tumors arising at other anatomical sites, and the neoplasm could be classified into classic and proximal subtypes. The classic subtype was more common (5), as observed in our case, which featured multinodular or granuloma-like architectures often accompanied by central necrosis or hyalinization. Tumor cells displayed biphasic differentiation, with admixed epithelioid and spindle cells that exhibited transitional morphology (13, 14). The epithelioid cells contained abundant eosinophilic cytoplasm and prominent nucleoli. The proximal subtype more frequently presented as large sheets or solid nests of large epithelioid cells with marked cytological atypia, and often contained rhabdoid cells with eosinophilic cytoplasmic inclusions (5, 6).

Immunohistochemical analysis was critical for the diagnosis of epithelioid sarcoma, and the typical immunoprofile encompassed three key features. Firstly, tumor cells exhibited co-expression of epithelial and mesenchymal markers, testing positive for CK-pan and EMA (epithelial markers) as well as Vimentin (mesenchymal marker). Secondly, CD34 positivity was detected in 50%–70% of cases, serving as a useful suggestive marker despite lacking specificity (15). Most importantly, loss of nuclear INI1 (*SMARCB1*) expression represented the most valuable and specific diagnostic marker; over 90% of epithelioid sarcomas harbored inactivating mutations or deletions of the *SMARCB1* gene, resulting in loss of INI1 protein expression in tumor cell nuclei, while surrounding normal cells retained positive staining and acted as a reliable internal control. This signature finding constituted the cornerstone for distinguishing epithelioid sarcoma from its histological mimics (13).

In this case, although immunohistochemistry showed complete loss of INI1 expression, the FISH analysis did not detect a classical homozygous deletion (0R2G) but rather a 1R(dim)1G pattern suggestive of a structural partial deletion. This discordance between IHC and FISH is not uncommon and can be explained by the molecular heterogeneity of *SMARCB1* alterations. Studies have shown that while homozygous deletion is the most frequent mechanism (approximately 75% of cases), other mechanisms include intragenic deletions, point mutations, and epigenetic silencing, all of which can lead to loss of protein expression without complete FISH-detectable deletion of both alleles (16, 17). In a large series, only 48% of tumors with *SMARCB1* loss by IHC showed complete deletion by FISH, emphasizing that FISH-negative cases with retained signals may still harbor cryptic alterations that disrupt gene function (18). Therefore, the combined IHC and FISH findings in our case are entirely consistent with the diagnosis of epithelioid sarcoma and illustrate the diversity of *SMARCB1* inactivation mechanisms.

Recent molecular studies demonstrated that classic and proximal subtypes were not merely morphological variants, but also molecularly distinct entities. Sigalotti et al. (19) identified that proximal-type epithelioid sarcoma was characterized by overactivation of the GATA3 and MYC pathways, coupled with epigenetic remodeling mediated by EZH2 overexpression. In contrast, classic-type epithelioid sarcoma exhibited an endothelial-like molecular profile, with expression of angiogenesis-related genes and activation of the SOX17 pathway. These findings provided a molecular basis for the biological differences between the two subtypes, and offered novel insights into subtype-specific diagnostic markers (e.g., GATA3 for proximal type, SOX17 for classic type) and targeted therapeutic strategies.

### 3.3 Differential diagnosis

In the present case, H&E staining revealed multinodular growth, central necrosis, a granuloma-like appearance, and a biphasic pattern composed of epithelioid and spindle cells. These morphological features raised an initial suspicion of epithelioid sarcoma. Prior to immunohistochemical testing, we systematically evaluated other epithelioid tumors that may arise in the orbit according to morphological characteristics. First, poorly differentiated carcinoma, including squamous cell carcinoma, adenocarcinoma and salivary gland-type neoplasms, needs to be excluded with the aid of clinical information to rule out metastatic lesions. Second, lesions with a highly vascular stroma and imaging findings indicative of a vascular neoplasm should be differentiated from epithelioid angiosarcoma. Third, tumors characterized by prominent nucleoli are suggestive of melanoma. Fourth, lesions showing multinodular architecture combined with mixed epithelioid and spindle cell morphology may represent epithelioid malignant peripheral nerve sheath tumor or biphasic synovial sarcoma. Fifth, epithelioid rhabdomyosarcoma should be considered in young patients when tumor cells contain abundant eosinophilic cytoplasm and prominent nucleoli. This stepwise morphological assessment guided the selection of immunohistochemical markers. We initially adopted a limited antibody panel because the patient received outpatient care, and all immunohistochemical results are summarized in **Table 1**.

The orbit is anatomically complex, and epithelioid sarcoma occurring at this site is extremely rare. Therefore, a rigorous differential diagnosis is essential. **Table 3** lists the key distinguishing characteristics between epithelioid sarcoma and its histological mimics. Poorly differentiated carcinoma, whether primary or metastatic, is relatively common in the orbit. Its immunophenotype varies depending on the primary origin, as reflected by markers such as CK7, CK20, p40 and TTF-1. Tumor cells of poorly differentiated carcinoma typically do not co-express vimentin and CD34, and maintain normal INI1 expression. A thorough review of clinical history facilitates accurate differentiation. Amelanotic melanoma in particular expresses melanocytic markers including S-100, SOX10, HMB45 and MelanA, while it is generally negative for epithelial markers and preserves intact INI1 expression (20). Epithelioid angiosarcoma is positive for cytokeratin and CD34, and consistently exhibits strong immunoreactivity for CD31 and ERG alongside retained INI1 expression (13, 21). In our case, the tumor was negative for CD31 and ERG and showed complete loss of INI1 expression, which ruled out epithelioid angiosarcoma. Biphasic synovial sarcoma may form glandular structures within its epithelioid component. It is positive for cytokeratin, EMA and, TLE1, but negative for CD34, and is defined by the t(X;18) translocation and SS18-SSX gene fusion. INI1 expression remains intact in this tumor (22). Epithelioid malignant peripheral nerve sheath tumor shows strong positive expression of S-100, SOX10 is usually positive, and CK and EMA can be focally positive. Approximately half of these cases are associated with NF1 gene mutation (23). Epithelioid rhabdomyosarcoma expresses MyoD1, myogenin and desmin with preserved INI1 expression (20). In contrast, epithelioid sarcoma is positive for cytokeratin and EMA and presents with loss of INI1 expression (24).

**Table 3:** Differential diagnosis of epithelioid sarcoma versus other epithelioid orbital tumors.

Entity	Morphology	Immunophenotype	Molecular/genetic features
Epithelioid sarcoma	Multinodular growth, central necrosis, granuloma-like appearance, biphasic epithelioid and spindle cells	CK+, EMA+, Vimentin+, CD34+, INI1–	Loss of SMARCB1 function
Poorly differentiated carcinoma (primary or metastatic)	Variable architecture, predominantly glandular or solid; squamous or salivary gland-type may be present	CK+, lineage-specific markers (CK7/CK20, p40, TTF-1, etc.)+; Vimentin–, CD34–, INI1+	Dependent on primary site and tumor type
Malignant melanoma (amelanotic subtype)	Epithelioid or spindle cells with prominent nucleoli	S100+, SOX10+, HMB45+, MelanA+, CK–, INI1+	BRAF, NRAS and other mutations
Epithelioid angiosarcoma	Epithelioid cells, vascular channels, hemorrhage	CK+, CD34+, CD31+, ERG+, INI1+	–
Biphasic synovial sarcoma	Epithelioid and spindle cells with distinct glandular lumina	CK+, EMA+, TLE1+, CD34–; INI1+ (partial loss possible)	t(X;18), SS18-SSX1/2 fusion
Epithelioid malignant peripheral nerve sheath tumor (MPNST)	Multinodular growth, epithelioid cells, may show wavy nuclei	S100+ (diffuse strong), SOX10+; CK(focal), EMA(focal); INI1+ (loss may occur in rare cases)	NF1 mutation
Epithelioid rhabdomyosarcoma	Small round blue cells, rhabdomyoblasts, epithelioid morphology	MyoD1+, myogenin+, desmin+, INI1+	TP53 and other fusions

In summary, INI1 loss is the key diagnostic marker of epithelioid sarcoma, helping to exclude tumors with retained INI1 expression (e.g., epithelioid angiosarcoma, melanoma, rhabdomyosarcoma) and to distinguish entities with partial INI1 loss (e.g., synovial sarcoma). It is particularly valuable in differentiating epithelioid sarcoma from malignant rhabdoid tumor (24).

### 3.4 Treatment and prognosis

Due to the rarity of Primary Epithelioid Sarcoma of Orbit, no standardized treatment protocol had been established. Based on the treatment principles for soft tissue sarcomas and limited case experience, wide and complete radical resection (R0 resection) was the cornerstone of potential cure. However, the confined orbital space adjacent to the optic nerve and extraocular muscles often rendered wide resection challenging, leading to a high local recurrence rate. The role of postoperative adjuvant radiotherapy had gained increasing recognition. According to the case comparisons in **Table 2**, patients treated with surgery combined with radiotherapy (cases 5, 6 and the present case) achieved favorable local control, suggesting that radiotherapy helped compensate for insufficient resection margins and reduce the risk of local recurrence. Modern techniques such as IMRT and proton therapy improved the protection of surrounding normal tissues. For unresectable, advanced or metastatic patients, chemotherapy represented a therapeutic option but showed limited efficacy. Recently, targeted therapy had achieved breakthrough progress. Loss of INI1 led to hyperactivation of the histone methyltransferase EZH2, which promoted tumor progression. Accordingly, the EZH2 inhibitor tazemetostat was approved by the US FDA in 2020 for the treatment of unresectable, metastatic or locally advanced epithelioid sarcoma, providing a novel systemic therapeutic option (25).

The prognosis of primary orbital epithelioid sarcoma was associated with multiple factors. A recent large-scale analysis of 1123 patients revealed that the stage at diagnosis was the most critical prognostic factor (distant metastasis conferred a 5.379-fold higher risk of death compared with localized disease). Each 1-cm increase in tumor size elevated the mortality rate by 7.8%, and surgical resection reduced mortality by 60.3% (2). The study also reported that the 5-year cancer-specific survival rate reached 53.7% and the median overall survival was 84 months in the modern treatment era. Prognosis also correlated with histological subtype: the proximal subtype (cases 3, 4, 6) tended to have a poorer prognosis, with early recurrence or metastasis. Long-term close follow-up was therefore essential.

### 3.5 Limitations

We acknowledge several limitations. First, as a single case report, the findings may not be generalizable to all patients. Second, the follow-up period is short (7 months), which limits assessment of long-term recurrence or metastasis. We have initiated regular surveillance in accordance with NCCN guidelines (11, 12) and will report outcomes in future updates. Despite these limitations, the extreme rarity of this entity and the first documentation in Asia justify reporting.

## 4 Conclusions

Primary epithelioid sarcoma of orbit poses substantial diagnostic challenges in both clinical and pathological settings. Non-specific clinical features frequently result in delayed diagnosis. Pathologists should recognize its characteristic multinodular morphology and biphasic epithelial-mesenchymal immunophenotype, and regard INI1 loss as the core diagnostic criterion, accompanied by systematic differential diagnosis. Recent molecular studies uncover molecular heterogeneity between the classic and proximal subtypes, opening new avenues for subtype-specific diagnosis and targeted therapy (19). Treatment follows a multidisciplinary model centered on wide surgical resection, with active consideration of postoperative adjuvant radiotherapy. Based on large population-based studies, surgical resection and early diagnosis are the key to improving prognosis (2). With the application of targeted agents such as tazemetostat, the prognosis of advanced-stage patients improves. Enhanced awareness and vigilance among multidisciplinary specialists are essential for early diagnosis, precise treatment, and ultimately better clinical outcomes for patients.

## Data Availability

The original contributions presented in the study are included in the article/supplementary material. Further inquiries can be directed to the corresponding author.
